# Diet and Oviposition Deprivation Effects on Survivorship, Gonotrophic Dissociation, and Mortality of *Anopheles gambiae* s.s.

**DOI:** 10.1155/2022/6313773

**Published:** 2022-06-18

**Authors:** Paulo S. Chisulumi, Bahati Nampelah, Revocatus Yohana, Anitha Philbert, Eliningaya J. Kweka

**Affiliations:** ^1^Department of Zoology and Wildlife Conservation, College of Natural and Applied Sciences, University of Dar es Salaam, Dar es Salaam, Tanzania; ^2^Department of Medical Parasitology and Entomology, School of Medicine, Catholic University of Health Sciences, P.O. Box 1464, Mwanza, Tanzania; ^3^Tropical Pesticides Research Institute, Division of Livestock and Human Disease Vector Control, Mosquito Section, P.O. Box 3024, Arusha, Tanzania

## Abstract

Diet quality is of paramount importance for egg batch size, longevity, and mortality of vector mosquitoes. Oviposition site presence and absence assumed to be dry season means a lot to the survivorship and mortality of most anthropophilic malaria vectors in sub-Saharan Africa. This study has assessed the effect of different diets and oviposition-site deprivation (OSD) on survivorship, longevity, and mortality of *An. gambiae* s.s. To determine the effect of diet and OSD on mortality, gonotrophic dissociation rates, longevity, and survivorship, six treatments were employed: Blood Fed with Oviposition (BFO), Blood Fed without oviposition (BF), Blood and Sugar Fed with Oviposition (BSFO), Blood and Sugar Fed without oviposition (BSF), Sugar Fed with Oviposition (SFO), and Sugar Fed without oviposition (SF). Mortality and gonotrophic dissociation were monitored daily. This study found that female mosquitoes offered blood meals with sugar solution and oviposition deprivation survived longer than those deprived of oviposition deprivation. Similarly, female mosquitoes fed on blood and provided with oviposition deprivation lived longer than those without oviposition deprivation. The gonotrophic dissociation rates were found to be lower in groups provided with oviposition deprivation. Our results show that OSD has a direct impact on the survivorship, gonotrophic dissociation rate, and longevity of the malaria anthropophilic vector, *An. gambiae* s.s., regardless of the diet.

## 1. Introduction

*Anopheles gambiae* s.l. are the main malaria vectors across sub-Saharan Africa which need oviposition sites, blood meal, and successful breeding [1–4] for survivorship and reproduction. In the dry season of the year, habitats are unstable; hence, mosquitoes suffer from oviposition-site deprivation (OSD). The effects of OSD, blood meals, and nectar (sugar) availability are of paramount importance in vector survivorship, fitness, and reproduction [1–4]. In the onset of extended drought, mosquitoes must ensure their survivorship to the productive habitats that are available [5–8]. It is assumed that the deprivation of an oviposition site can play a role in extending survivorship by changing the physiological response in reproduction cycles [4, 9]. Malaria parasite transmission by mosquitoes is a function of several parameters such as longevity, gonotrophic dissociation, food type taken by mosquito vector, and host vector-contact [10]. A life table is a means of describing mosquito longevity and other vital events in the population; however, the average longevity of flies in captivity is less than under suitable environmental conditions, although females of the hibernating species may live for longer [11]. The longevity of female mosquitoes determines the vectorial capacity; mosquitoes that live long will have several opportunities to transmit malaria parasites [12]. Furthermore, a condition when the eggs develop after blood meal has been ingested and there is failure to be laid is termed as the gonotrophic dissociation and they are used as a source of energy for survival after a successful blood meal has been taken by mosquitoes [13, 14]. This concept is important as it reduces vector density which translates to reduced malaria cases. However, food is essential to mosquito longevity and egg development, blood meal is used to provide protein that is important for the development of eggs, and sugar meal is used to provide energy for flight survival, improving fecundity and finding oviposition deprivation for laying eggs [1, 15]. Moreover, oviposition deprivation is defined as an absence of a potential oviposition sites which causes mosquitoes to retain eggs instead of laying them [16]. Oviposition deprivation or larval habitat deprivation is a condition that occurs when mosquitoes develop eggs without laying them [17]. Oviposition deprivation differs from egg retention which occurs when the female mosquito does not lay (some) developed eggs despite having access to a suitable oviposition [17]. Other studies have shown low effects of OSD to *Ae. aegypti* and *Cx. fatigans* than could be similar to anopheline species [18–20], but all had reduced reproductive output having reduced egg batch size [18]. These effects were directly proportional to the number of days mosquitos were exposed to OSD [18]. Supplements such as sugar have been found to have the influence on increasing hatchability [3].

The availability of blood meals and OSD has not been assessed well on gonotrophic dissociation and survivorships in other species. In this study, we hypothesize that OSD reduces gonotrophic dissociation and increases survivorship of mosquitoes by avoiding the costs associated with oviposition and energy loss [5, 21].

There is limited information about the impact of diet and oviposition-site deprivation on the longevity, gonotrophic dissociation, and mortality rates of *Anopheles gambiae* s.s. Therefore, the present study is aimed at assessing the impact of diet and oviposition deprivation on the longevity, gonotrophic dissociation, and mortality rates of *Anopheles gambiae* s.s.

## 2. Methods and Materials

### 2.1. Mosquito Rearing

Mosquitoes were reared under standard insectary conditions (temperature 27 ± 2°C, relative humidity (RH) 75% ± 2%, and photoperiod L : D 12 : 12 hours) [22]. The adult mosquitoes were fed on blood from the pet rabbit host for one hour until fully engorged and left to lay eggs in moist white filter paper for 1-2 days; eggs were then transferred to the desiccator for 2-3 days for maturation. The eggs were allowed to hatch in plastic basins containing 1.5 L of dechlorinated rain water. Hatched larvae were fed on Tetramin fish food 0.0003 mg per larvae until they metamorphosed to the pupa stage [22, 23]. The pupae were transferred to the mosquito cage until emergence. Adult mosquitoes were fed with 10% sucrose solution daily.

### 2.2. Experimental Design

**Figure 1:**
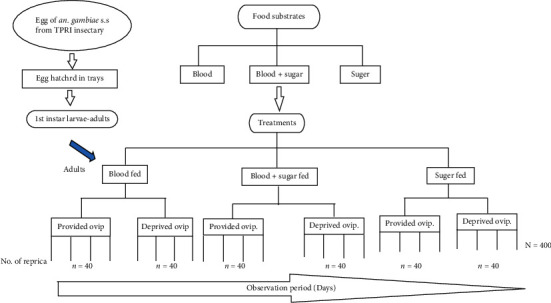
Summary of the experimental study design.

A cohort of 400 adult *Anopheles gambiae* s.s. emerging from the pupae of the laboratory colony was fed for a duration of 3 days with a 10% sucrose solution soaked in cotton wool. The cotton wool was soaked in fresh sucrose solution daily and put on the top of the cage. Ten (10) three- (3-) day-old females were selected randomly then transferred to the mosquito's cage, and the treatment was started. For the blood-feeding individuals, the sugar solution was taken out 2 hours before feeding them. The blood meal was offered for 1 hour and subsequently once after every 4 days to the live rabbit host until they were fully engorged. This was repeated until the mosquitoes in the treatment arm died. The live animals (pet, rabbit host) enclosed in net material in boxes were put inside the mosquito's cages to allow the mosquitoes to feed the blood. For each treatment, there were 4 replicates as follows: (i) mosquito fed on sugar with and without oviposition site, (ii) mosquito fed on blood with and without oviposition site, and (iii) mosquito fed on both sugar and blood with and without oviposition site, as shown in Figure 1. Sugar solution was changed daily in the appropriate treatments. If the experimental mosquito died before the lapse of two days, it was replaced by another female mosquito from the same cohort left for replacement purposes, every day (after every 24 hours); all live mosquitoes were recorded. The number of blood-fed females was checked after providing them with the blood meal and half-gravid (or gravid) females were recorded daily. The eggs laid daily were recorded.

There were two cages for each treatment, the first cage was provided with the oviposition sites, and the second was deprived of the oviposition sites. Three-day-old females were selected randomly and transferred to the mosquito cage by using an aspirator and then were fed with a live pet rabbit host continuously in the interval of 4 days. In this set up, a total of 240 female adults of *An. gambiae* s.s. were used to record mortality and to monitor longevity at the same time and were recorded after every 24 hours.

### 2.3. Determination of the Effect of Diet and Oviposition Deprivation on the Longevity and Mortality Rates of *Anopheles gambiae* s.s.

The longevity and mortality rate (number of dead mosquitoes/number of mosquitoes of *Anopheles gambiae* s.s.) was assessed from the first meal until death of mosquito. A total of ten (10) female mosquitoes were chosen and treated until they died naturally.

### 2.4. Examining the Effect of Diet and Oviposition Deprivation on the Gonotrophic Dissociation Rates of Gravid *Anopheles gambiae* s.s.

The mosquito gonotrophic dissociation was assessed after eggs have been developed by *Anopheles gambiae* s.s. The adult and female mosquitoes were allowed to mate with males for three days after emergence; then, ten (10) females were sorted randomly from males by using an aspirator and allowed to feed on blood then followed up until the gonotrophic dissociated naturally. There were two cages, the first cage was provided with oviposition deprivation and the second without oviposition deprivation for each treatment. A total of 160 female adult *An. gambiae* s.s. were used for these sets to monitor gonotrophic dissociation time. The mosquitoes were blood fed once throughout the experiment, but for those fed on both blood and sugar meals, a sugar solution was offered daily. Female mosquitoes were considered gravid when their abdomens were wider and whitish; on the other hand, they were considered not gravid when their abdomens were slimy. The presence of gravid or half-gravid female mosquitoes was quantified by actual counting with the aid of a hand lens and was recorded every 24 hours.

### 2.5. Data Analysis

Data were entered in Microsoft Excel 2019 (Microsoft, WA, USA) for validation and summarization, then transferred to SPSS program for Windows version 25.0 (IBM Corp., Armonk, NY, USA) for analysis. One-way analysis of variance (ANOVA) and post hoc test (Tukey HSD test for one-way ANOVA) were performed to assess the impact of food type (blood/sugar) and oviposition deprivation (with or without) on longevity, gonotrophic dissociation, and mortality of *An. gambiae* s.s. A significance level of 0.05 was used to confirm the association between independent and dependent variables. Results were presented in tables and graphs. The Kaplan-Meier survival curves were used to compare longevity of *An. gambiae* s.s. fed with blood sugar and provided or deprived with oviposition deprivation.

## 3. Results

### 3.1. The Effects of Diet and Oviposition Site on the Longevity of *Anopheles gambiae* s.s.

**Figure 2:**
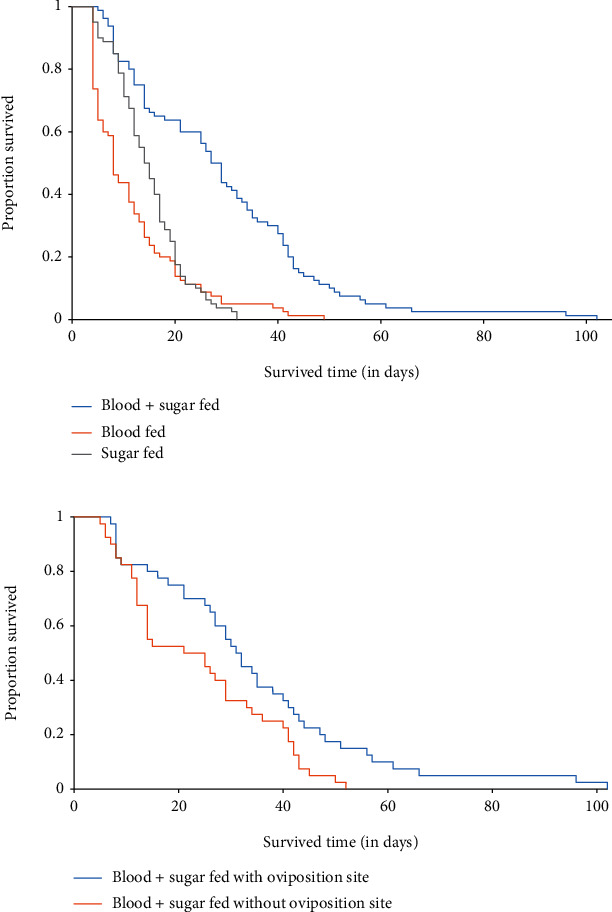
(a) Kaplan-Meier survival curve of adult *An. gambiae* s.s. fed on both blood and sugar, blood alone, and sugar alone provided and deprived oviposition sites. (b) Kaplan-Meier survival curve of adult *An. gambiae* s.s. fed on both blood and sugar provided and deprived oviposition site.

**Figure 3:**
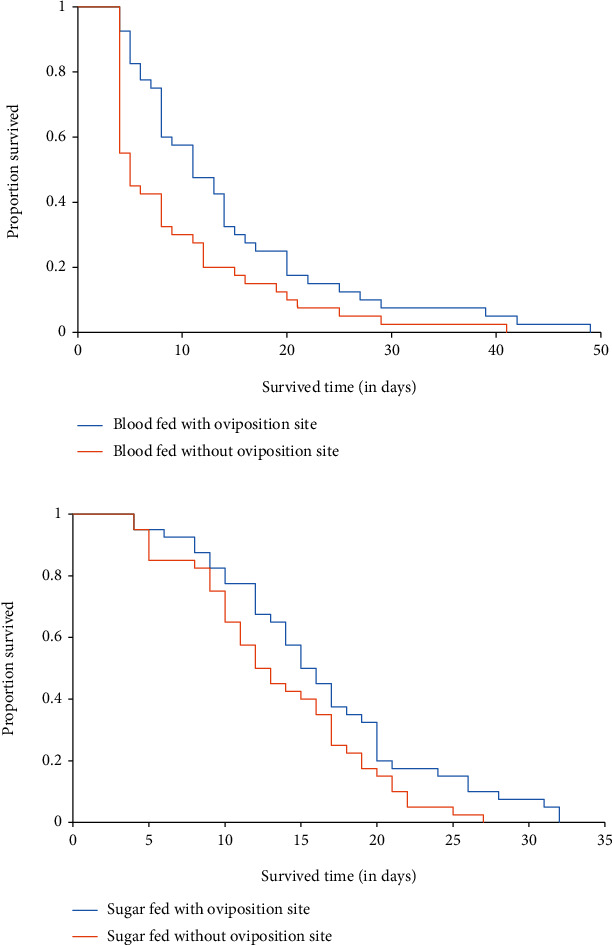
(a) Kaplan-Meier survival curve of adult *An. gambiae* s.s. fed on blood alone provided and deprived oviposition site. (b) Kaplan-Meier survival curve of adult *An. gambiae* s.s. fed on sugar alone provided and deprived oviposition site.

The results of this study found that female *An. gambiae* s.s. offered both blood and sugar survived longer than those fed on blood alone and sugar alone, which was statistically significant (*F* = 10.491; df = 5; *P* < 0.001) (Figure 2(a)). Female *An. gambiae* s.s. fed on both blood and sugar provided with oviposition deprivation survived longer than those fed on both blood and sugar deprived with oviposition deprivation, which was statistically significant (*P* = 0.009) (Figure 2(b)). Female *An. gambiae* s.s. fed on blood alone provided with oviposition deprivation survived longer than those fed on blood alone deprived with oviposition deprivation, which was not statistically significant (*P* = 0.765) (Figure 3(a)). Female *An. gambiae* s.s. fed on sugar alone with oviposition deprivation survived longer than those fed on sugar alone without oviposition deprivation, which was not statistically significant (*P* = 0.996) (Figure 3(b)).

### 3.2. The Effect of Diet and Oviposition Deprivation on the Gonotrophic Dissociation Rates of *Anopheles gambiae* s.s.

**Figure 4:**
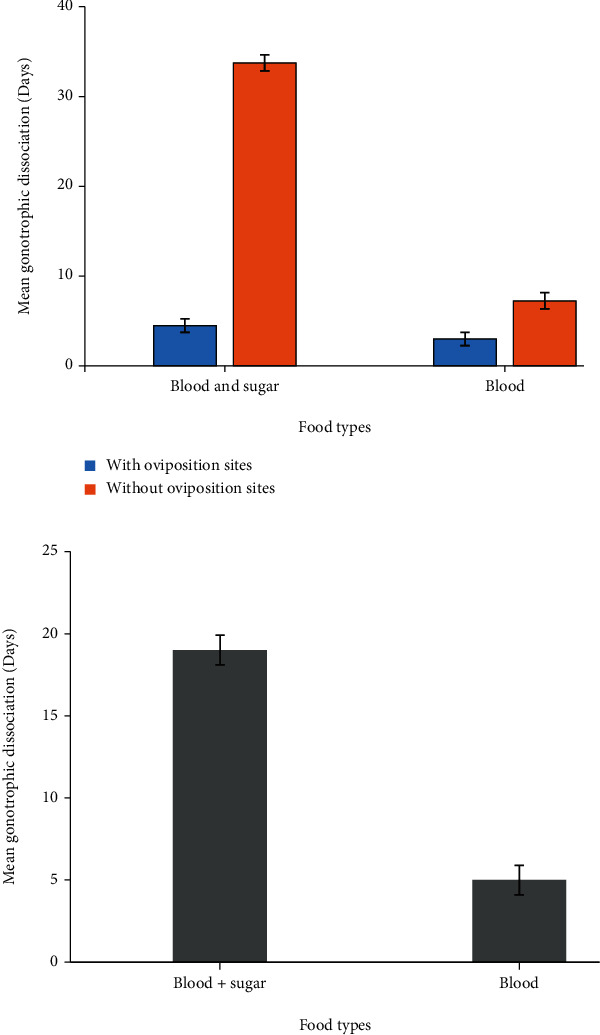
(a) The mean gonotrophic dissociation time of gravid *An. gambiae* s.s. fed on both blood and sugar and blood alone with or without oviposition deprivation. (b) The mean gonotrophic dissociation time of gravid *An. gambiae* s.s. fed on both blood and sugar and blood alone, with or without oviposition deprivation.

This study found that female *An. gambiae* s.s. fed on both blood and sugar had longer gonotrophic dissociation time than those fed on blood alone and sugar alone, which was statistically significant (*F* = 102.792; df = 3; *P* < 0.001) (Figure 4(a)). Female *An. gambiae* s.s. fed on both blood and sugar solution deprived oviposition deprivation had a longer gonotrophic dissociation time than those fed on both blood and sugar provided with oviposition deprivation, which was statistically significant (*P* < 0.001). Female *An. gambiae* s.s. fed on blood alone deprived of oviposition deprivation had a longer gonotrophic dissociation time compared to those fed on blood provided with oviposition deprivation, which was statistically not significant (*P* = 0.879) (Figure 4(b)).

### 3.3. The Effect of Diet and Oviposition Site on the Mortality Rates of *Anopheles gambiae* s.s.

**Figure 5:**
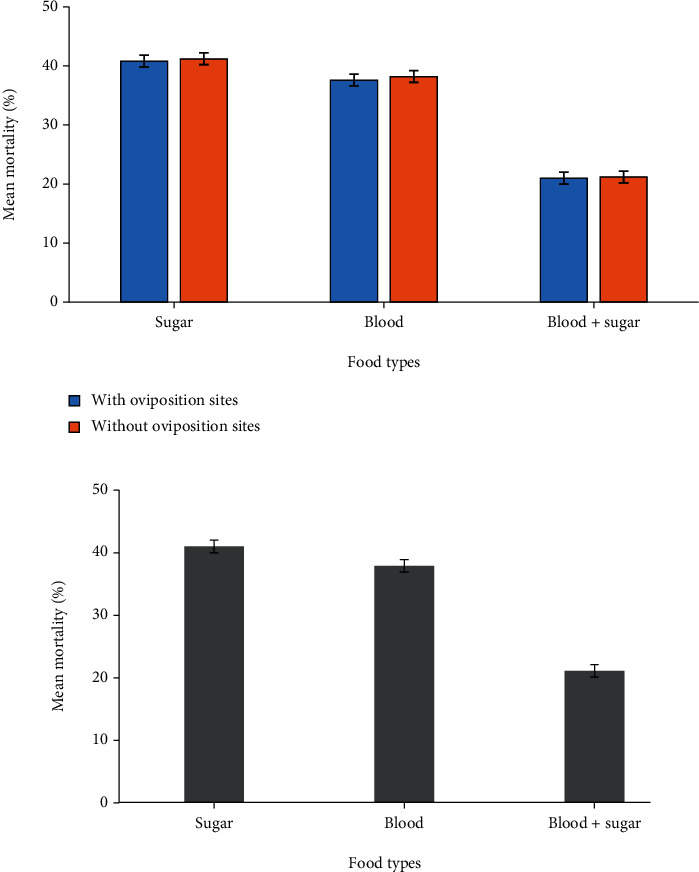
(a) The mean mortality of *An. gambiae* s.s. fed on both blood and sugar, blood alone, and sugar alone provided or without oviposition deprivation. (b) The mean mortality of *An. gambiae* s.s. fed on both blood and sugar, blood alone, and sugar alone and provided or without oviposition deprivation.

Mortality is significantly influenced by diet; however, this study shows that mortality was higher in *An. gambiae* s.s. fed on sugar alone than those fed on both (blood+sugar) and blood alone, which was not statistically significant (*F* = 2.164; df = 5; *P* = 0.104) (Figure 5(a)). Female *An. gambiae* s.s. fed on both blood and sugar provided with oviposition deprivation had the same mortality to those fed on both blood and sugar deprived oviposition deprivation, which was statistically not significant (*P* = 0.994) (Figure 5(b)). Female *An. gambiae* s.s. fed on blood alone provided oviposition deprivation had the same mortality to those fed on blood alone deprived oviposition deprivation, which was statistically not significant (*P* = 1.000) (Figure 5(b)). Female *An. gambiae* s.s. fed on sugar alone provided with oviposition deprivation had the same mortality to those fed on sugar alone deprived of oviposition deprivation, which was statistically not significant (*P* = 0.999) (Figure 5(b)).

## 4. Discussion

The findings of this study have shown that mean longevity ranged between 26 and 47 days while gonotrophic dissociation ranged from 2 to 41 days depending on the treatment and conditions availed to *An. gambiae* s.s. The food source and availability of habitat play a major role in longevity, gonotrophic dissociation, and adult mortality. This means that when mosquitoes have access to both host (blood) and plant nectar (sugar), they have a great chance to survive longer compared to those fed on the host alone and plant nectar alone because both plant nectar and mosquito host provide more energy and protein necessary for mosquito oviposition and longevity. Host blood and plant nectars increase the longevity of mosquitoes, thus leading to increased malaria vector reproduction capacity (population) and the chance of malaria vectors to transmit malaria pathogens. Similar findings were previously reported in *An. gambiae* s.s. and *Aedes aegypti* [24, 25].

The findings of this study further demonstrated that when oviposition deprivation was provided to *Anopheles gambiae* s.s. fed with blood meals in combination with sugar meals lived longer than mosquitoes which were not provided with oviposition deprivation when fed with blood meals in four days intervals and sugar solution fed daily. These findings are similar to previous studies which found that female *Anopheles gambiae* when offered oviposition deprivation and a blood meal with sugar solution daily survived longer than mosquitoes fed on blood meal alone [2]. Furthermore, other studies showed that many anautogenous female mosquito species survived longer when they were fed on vertebrate blood with sugar meals [1, 15, 26, 27] than those fed daily on blood meals alone [1, 28]. Additionally, the findings on longevity are similar to the study by Kessler and others [29, 30] whose findings showed that female mosquitoes fed on sugar have a maximum lifespan of 24 days, while [28] reported that the lifespan of *Anopheles gambiae* fed on sugar only was 20 days. These studies have shown that the blood meal is an important parameter in the production of eggs and survivorship of mosquito, but nectary is an additional energy source which adds value in all life-history traits. This study implies that when mosquitoes are deprived of oviposition deprivation such as fresh or salt-water marshes, grassy ditches, rice fields, mangrove swamps, small temporary rain ponds, and edges of streams and rivers, they can live shorter and they can have small chances to increase population and transmit malaria pathogen. This can be a strategy to control malaria vectors by reducing mosquito reproduction capacity and the possibilities of transmitting malaria pathogens. However, the longer a female mosquito survives, the higher the risk it poses in terms of malaria transmission [31, 32].

The findings of this study on mean gonotrophic dissociation time in a laboratory population of *Anopheles gambiae* s.s. indicated the occurrence of this phenomenon with the influence of diet and oviposition deprivation. The blood meal in combination with sugar solution and blood meals alone provided with or without oviposition deprivation evoked different mean gonotrophic dissociation times of *Anopheles gambiae* s.s.

The female *Anopheles gambiae* s.s. fed on both blood meals and sugar solution has longer mean gonotrophic dissociation time than those fed on blood alone and sugar alone. This means that gravid mosquitoes fed on both blood and sugar gonotrophic take a long time to dissociate compared to those fed on blood alone which take a short period because both blood and sugar influencing mosquitoes had large egg batch size which dissociates for a long period compared to those fed on blood alone. Additionally, mosquitoes fed on both blood and sugar had slow egg dissociation time because they had sufficient reserves of sugar and protein, so they do not consume/dissolve their eggs as a source of energy for survival. A study was conducted to assess whether gonotrophic dissociation among female *Ae. albopictus* fed on chicken blood, guinea pig blood, and human blood occurs, reporting that multiple blood meals caused the occurrence of gonotrophic dissociation to decrease similar to our findings [14]. Additionally, gonotrophic dissociation has been reported in field populations of *Anopheles* and *Culex* mosquitoes in which the concordant female develops more fat tissues that those undergoing dissociations [14, 33].

This result is supported by a study that reported that when oviposition deprivation is deprived, female mosquitoes, especially those with access to a blood meal, would live longer than nonoviposition deprivation deprived because they could potentially allocate more nutrients into maintenance and avoid the direct and indirect cost of reproduction (Artis et al. 2014). Gonotrophic dissociation has been reported in field populations of *Anopheles* and *Culex* mosquitoes [33], and these few studies prove that when female mosquitoes fed on blood, they develop eggs, but when the oviposition site is deprived, they dissolve their eggs which are used as a source of energy to increase the chance of survival. This concept of malaria vector control implies that when a gravid mosquito lacks suitable larva habitat or oviposition deprivation, they fail to lay eggs; instead, they dissolve and use it as a source of energy for survival. This can be used as an intervention to control the malaria vector population and their associated diseases.

In this study, it seems that for *Anopheles gambiae* s.s. after being fed with different diets provided or deprived with oviposition deprivation, mortality rates were higher in mosquitoes that fed on sugar alone followed by mosquitoes fed on blood meal alone. However, mortality rates were very low in mosquitoes fed on blood meals with sugar because blood meal alone and blood meal in combination with sugar seemed to provide important protein and energy for survival and increase the longevity of mosquitoes. This means that when infected female mosquitoes are deprived of blood meal sources by reducing vector contact to the host by using mosquito repellents and insecticide-treated nets, they live a shorter time and have small chances to transmit the malaria pathogens. This can be used as a strategy for reduce the reproduction capacity of malaria vectors and the chances of transmitting malaria pathogen [31]. Nevertheless, the oviposition site is noted to have no effect on the mortality rates of *Anopheles gambiae* s.s. The results of this study are supported by a study conducted by Neto and Navarro-Silva (2002) on *Aedes albopictus* which shows that the mortality rate of adult mosquitoes provided with or deprived with oviposition deprivation was constant throughout[34].

## 5. Conclusion

This study shows that diet (blood+sugar) and deprivation of oviposition deprivation have a significant effect on malaria vectors: the lifespan of *An. gambiae* s.s. when fed on blood followed by sugar solution meals and deprived oviposition deprivation was significantly reduced compared to that of their counterparts. The findings are important in the control of malaria because the short lifespan of vectors translates to reduced malaria transmission cases. These concepts should be explored further for possible scale-up to complement the existing malaria control interventions.

## Data Availability

All data associated with this manuscript are within the manuscript discussed herewith.

## References

[1] Gary R. E., Foster W. A. (2001). Effects of available sugar on the reproductive fitness and vectorial capacity of the malaria vector Anopheles gambiae (Diptera: Culicidae). *Journal of Medical Entomology*.

[2] Gary R. (2005). *Biology of the Malaria Vector Anopheles Gambiae: Behavioral and Reproductive Components of Sugar Feeding*.

[3] Manda H., Gouagna L. C., Foster W. A. (2007). Effect of discriminative plant-sugar feeding on the survival and fecundity of Anopheles gambiae. *Malaria Journal*.

[4] Dieter K. L., Huestis D. L., Lehmann T. (2012). The effects of oviposition-site deprivation on Anopheles gambiae reproduction. *Parasit Vectors*.

[5] Dao A., Kassogue Y., Adamou A. (2010). Reproduction-longevity trade-off in Anopheles gambiae (Diptera: Culicidae). *Journal of Medical Entomology*.

[6] Lehmann T., Dao A., Adamou A. (2010). Aestivation of the African malaria mosquito, Anopheles gambiae in the Sahel. *The American journal of tropical medicine hygiene*.

[7] Omer S. M., Cloudsley-Thompson J. (1970). Survival of female Anopheles gambiae Giles through a 9-month dry season in Sudan. *Bulletin of the World Health Organization*.

[8] Omer S., Cloudsley-Thommson J. (1968). Dry season biology of Anopheles gambiae Giles in the Sudan. *Nature*.

[9] McDonald J. L., Lu L. C. (1972). Viability of mosquito eggs produced by female mosquitoes denied ovipositing sites. *Mosquito News*.

[10] Charlwood J. D., Nenhep S., Sovannaroth S. (2016). ‘Nature or nurture’: survival rate, oviposition interval, and possible gonotrophic discordance among South East Asian anophelines. *Malaria Journal*.

[11] Mautz B. S., Rode N. O., Bonduriansky R., Rundle H. D. (2019). Comparing ageing and the effects of diet supplementation in wild vs. captive antler flies, Protopiophila litigata. *Journal of Animal Ecology*.

[12] Macdonald G. (1957). The epidemiology and control of malaria. *The Epidemiology and Control of Malaria.*.

[13] Swellengrebel N. (1929). La dissociation des fonctions sexuelles et nutritives (dissociation gono-trophique) d ‘Anopheles maculipennis comme cause du paludisme dans les Pays-Bas et ses rapports avec—l ‘infection domiciliaire. *Annales de l'Institut Pasteur*.

[14] Xue R.-D., Barnard D. R., Ali A. (2009). Influence of multiple blood meals on gonotrophic dissociation and fecundity in Aedes albopictus. *Journal of the American Mosquito Control Association*.

[15] Nayar J., Pierce P. (1980). The effects of diet on survival, insemination and oviposition of Culex nigripalpus Theobald. *Mosquito News*.

[16] Herrera-Varela M., Lindh J., Lindsay S. W., Fillinger U. (2014). Habitat discrimination by gravid Anopheles gambiae sensu lato–a push-pull system. *Malaria Journal*.

[17] Yang P. (2008). *Effect of oviposition site deprivation on oviposition performance and egg hatch rate of naturally blood–fed gravid Culex quinquefasciatus (Diptera: Culicidae)*.

[18] El-Akad A., Humphreys J. (1988). Factors affecting oviposition and egg production in laboratory-reared anopheles pharoensis Theobald. *Bulletin of the Society of Vector Ecologists*.

[19] Wearne H. M., Shisler J. K. (1989). Stressed versus unstressed oviposition patterns in the saltmarsh mosquito, Aedes sollicitans (Walker)(Diptera: Culicidae). *Journal of the American Mosquito Control Association*.

[20] Briegel H. (2003). Physiological bases of mosquito ecology. *Journal of Vector Ecology*.

[21] Huestis D. L., Yaro A. S., Traoré A. I. (2011). Variation in metabolic rate of Anopheles gambiae and A. arabiensis in a Sahelian village. *Journal of Experimental Biology*.

[22] Kivuyo H. S., Mbazi P. H., Kisika D. S. (2014). Performance of five food regimes on Anopheles gambiae senso stricto larval rearing to adult emergence in insectary. *PLoS One*.

[23] Koenraadt C., Majambere S., Hemerik L., Takken W. (2004). The effects of food and space on the occurrence of cannibalism and predation among larvae of Anopheles gambiae s.l.. *Entomologia Experimentalis et Applicata*.

[24] Xue R. D., Ali A., Barnard D. R. (2005). Effects of forced egg-retention in Aedes albopictus on adult survival and reproduction following application of DEET as an oviposition deterrent. *Journal of Vector Ecology*.

[25] Huestis D. L., Lehmann T. (2014). Ecophysiology of _Anopheles gambiae s.l._ : persistence in the Sahel. *Infection, Genetics and Evolution*.

[26] Nayar J., Van Handel E. (1971). The fuel for sustained mosquito flight. *Journal of Insect Physiology*.

[27] Hancock R., Foster W. (1997). Larval and adult nutrition effects on blood/nectar choice of Culex nigripalpus mosquitoes. *Medical veterinary entomology*.

[28] Fernandes L., Briegel H. (2005). Reproductive physiology of Anopheles gambiae and Anopheles atroparvus. *Journal of Vector Ecology*.

[29] Kessler S., Vlimant M., Guerin P. M. (2013). The sugar meal of the African malaria mosquito Anopheles gambiae and how deterrent compounds interfere with it: a behavioural and neurophysiological study. *Journal of Experimental Biology*.

[30] Kessler S., Vlimant M., Guerin P. M. (2015). Sugar-sensitive neurone responses and sugar feeding preferences influence lifespan and biting behaviours of the Afrotropical malaria mosquito, Anopheles gambiae. *Journal of Comparative Physiology A*.

[31] César Murillo B., Consuelo Jaramillo S., Jaime Quintero C., Mario Suárez T. (1989). Biología de Anopheles (Kerteszia) neivai H., D. & K., 1913 (Diptera: Culicidae) en la Costa Pacifica de Colombia: IV - Estructura etárea y transmisión de malaria. *Revista de Saúde Pública*.

[32] Artis M. L., Huestis D. L., Lehmann T. (2014). The effects of oviposition-site deprivation on longevity and bloodfeeding rate in Anopheles gambiae. *Parasites & Vectors*.

[33] Washino R. K. (1977). The physiological ecology of gonotrophic dissociation and related phenomena in mosquitoes. *Journal of medical entomology*.

[34] Löwenberg Neto P., Navarro–Silva M. A. (2004). Development, longevity, gonotrophic cycle and oviposition of Aedes albopictus Skuse (Diptera: Culicidae) under cyclic temperatures. *Neotropical Entomology*.

